# Antimalarial Exposure Delays *Plasmodium falciparum* Intra-Erythrocytic Cycle and Drives Drug Transporter Genes Expression

**DOI:** 10.1371/journal.pone.0012408

**Published:** 2010-08-25

**Authors:** Maria Isabel Veiga, Pedro Eduardo Ferreira, Berit Aydin Schmidt, Ulf Ribacke, Anders Björkman, Ales Tichopad, José Pedro Gil

**Affiliations:** 1 Malaria Research Lab, Department of Medicine, Karolinska Institutet, Stockholm, Sweden; 2 Drug Resistance and Pharmacogenetics Group, Institute of Biotechnology and Bioengineering, Centre of Molecular and Structural Biomedicine, University of Algarve, Faro, Portugal; 3 Department of Microbiology, Tumor and Cell Biology (MTC), Karolinska Institutet, Stockholm, Sweden; 4 Institute of Biotechnology AS CR, Prague, Czech Republic; 5 Physiology Weihenstephan, Technical University Munich, Freising-Weihenstephan, Germany; 6 Laboratory of Molecular Anthropology and Health, Department of Anthropology, Binghamton University, Binghamton, New York, United States of America; Université Pierre et Marie Curie, France

## Abstract

**Background:**

Multi-drug resistant *Plasmodium falciparum* is a major obstacle to malaria control and is emerging as a complex phenomenon. Mechanisms of drug evasion based on the intracellular extrusion of the drug and/or modification of target proteins have been described. However, cellular mechanisms related with metabolic activity have also been seen in eukaryotic systems, e.g. cancer cells. Recent observations suggest that such mechanism may occur in *P. falciparum*.

**Methodology/Principal Findings:**

We therefore investigated the effect of mefloquine exposure on the cell cycle of three *P. falciparum* clones (3D7, FCB, W2) with different drug susceptibilities, while investigating in parallel the expression of four genes coding for confirmed and putative drug transporters (*pfcrt*, *pfmdr1*, *pfmrp1 and pfmrp2*). Mefloquine induced a previously not described dose and clone dependent delay in the intra-erythrocytic cycle of the parasite. Drug impact on cell cycle progression and gene expression was then merged using a non-linear regression model to determine specific drug driven expression. This revealed a mild, but significant, mefloquine driven gene induction up to 1.5 fold.

**Conclusions/Significance:**

Both cell cycle delay and induced gene expression represent potentially important mechanisms for parasites to escape the effect of the antimalarial drug.

## Introduction

*Plasmodium falciparum* malaria remains a major disease burden in the developing world [1], chemotherapy being the foremost available tool for its control. Efficacious malaria treatment is presently dependent on the efficacy of artemisinin combination therapy (ACT), the global replacement of the previous mainstays, chloroquine and sulfadoxine-pyrimethamine [1], [2]. Unfortunately, recent data suggests that *P. falciparum* resistance to ACT might be presently developing, both to its long half-life components but also – and worryingly – against the artemisinin based components [3], [4]. The latter phenomenon has been mainly defined as an *in vivo* significant decrease in parasite reduction rate, manifested clinically by markedly longer parasite clearance times [5]. The molecular basis of this phenomenon is unclear, starting from the fact that most of these observations are not associated with altered artemisinin IC_50s_ in vitro [3]. It is conceivable that this phenomenon is related with other observations by us [6] and others [7], [8] showing that antimalarials can interfere with the cell cycle development of the parasite, potentially constituting a general first step towards high grade resistance to antimalarial drugs.

Drug resistance has been widely associated with the action of efflux pumps, able to transport drugs out of the relevant cellular compartment, and thereby displacing them from their molecular targets [9]. This action is expected to be potentiated through an increase in the availability of transporter proteins through enhanced gene transcriptional activity. In *P. falciparum* two transporters have been robustly associated with antimalarial resistance: PfCRT (chloroquine resistance transporter), a drug metabolite superfamily transporter coded by the *pfcrt* gene (MAL7P1.27) [10], [11], and the Pgh (P-glycoprotein homologue, coded by *pfmdr1* (PFE1150w)) an ATP-binding cassette (ABC) transporter superfamily member [12]. Two other ABC transporters potentially associated with *P. falciparum* drug resistance can be added - the homologues of the multidrug resistance-associated protein (MRP) type [13], [14], [15] coded by the *pfmrp1* (PFA0590w) and *pfmrp2* (PFL1410c) genes. Polymorphisms in these drug transporters have been associated with altered *in vitro* and/or *in vivo* responses to ACT components [16], [17], [18], [19], [20], [21], indicating that their common action might constitute a basis for wide range multidrug resistance phenotypes able to manage the new combinational therapy challenges faced by the parasite. Besides single nucleotide polymorphisms (SNPs), enhanced *locus* expression can drive decreased drug sensitivity, a phenomenon well documented for *pfmdr1*, where gene duplication events are strongly associated to mefloquine (MQ) resistance [22], [23] and lumefantrine decreased sensitivity [24].

The synthetic arylaminoalcohol MQ constitutes one of the central partner drugs in artemisinin combination therapy, widely used in South East Asia and South America. In SE Asia, the *in vivo* delayed clearance has been observed not only in artesunate monotherapy explorative regimens, but also associated to the regular mefloquine-artesunate ACT [3].

We hypothesize that the observed clearance delay is associated with this phenomena, contributing for a survival advantage in a fraction of the parasite infection, by allowing an extended time for the induction and expression of key drug transporters, leading to a pivotal decreased intracellular drug exposure in the first phase of the treatment course.

Using MQ as a relevant and convenient reference ACT antimalarial, we have focused on monitoring changes in the cell cycle progression rate of three *P. falciparum* clones with different MQ susceptibilities, while in parallel detecting variations in transcript abundance of the *pfmdr1*, *pfcrt*, *pfmrp1* and *pfmrp2* genes.

## Results

### Parasites genetic characterization

We have studied in parallel three *P. falciparum* clones, one MQ sensitive (W2) and two (3D7 and FCB) with decreased sensitivity. The *pfmdr1*, *pfcrt*, *pfmrp1* and *pfmrp2* ORF were fully sequenced to determine the genetic variability between the clones. The three clones, show two different main *pfmdr1* polymorphisms: a single nucleotide polymorphism at amino acid position 86 (N86 in 3D7, 86Y in W2 and FCB) and gene copy number variation (W2 and 3D7 harbouring one copy [25], with FCB carrying two copies [23]). Numerous single nucleotide polymorphism differences were seen in the three remaining genes (GenBank accession numbers: GU797309, GU797310, GU797311, GU797312, GU797315, GU797316).

### *Plasmodium falciparum* cell cycle progression alterations driven by mefloquine

*In vitro* MQ 50% inhibitory concentration (IC_50_) of W2, 3D7 and FCB clones were in average from 6 independent assays 10±3.3nM, 50±16.9nM and 50±14.1nM respectively. As for IC_99_ the average results was 44±13.9nM, 146±36.1nM, 146±50.6nM for W2, 3D7 and FCB respectively. These concentrations were used for challenging the clones in the assay performed.

**Figure 1:**
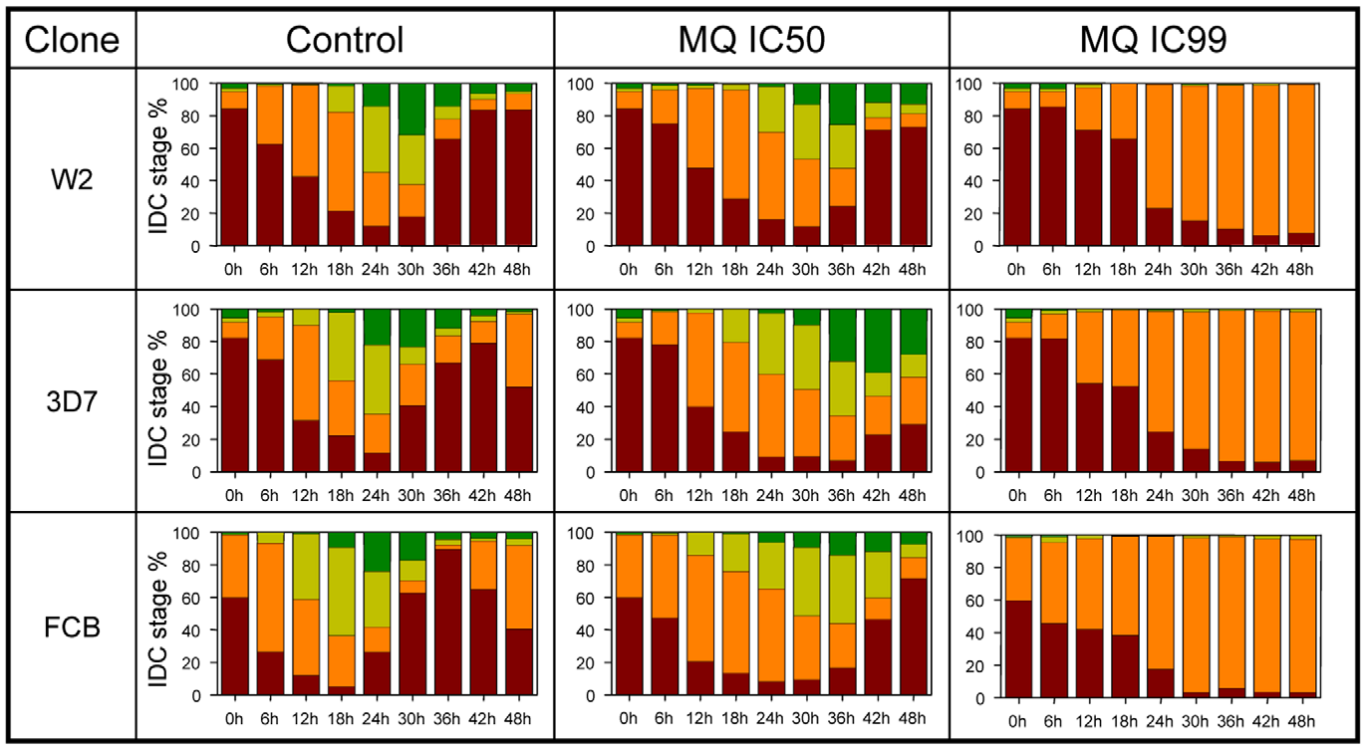
Stage morphology development throughout time upon MQ exposure. Parasite stage percentage (Y axis) of W2, 3D7 and FCB clones with continuous exposure at MQ IC_50_ and MQ IC_99_ throughout 48 hours time course (X axis). Four morphological stages were differentiated using light microscope: early rings (brown), late rings/early trophozoites (orange), trophozoites (light green) and schizonts (dark green).

Exposure to MQ induced an unexpected and unambiguous delay in the cell cycle progression of the three tested parasites, as evaluated by Giemsa staining under light microscopy for the four counted stages (Figure 1). The degree of delay effect was clone dependent.

At MQ IC_99_ a pronounced morphology arrest was seen in the three clones remaining essentially at the experimental initial ring stage with picnotic features. The quantification of end point RNA indicated the presence of viable cells, an observation supported for all tested parasites by their growth recovery 7–10 days after drug withdrawal (Figure S1).

**Figure 2:**
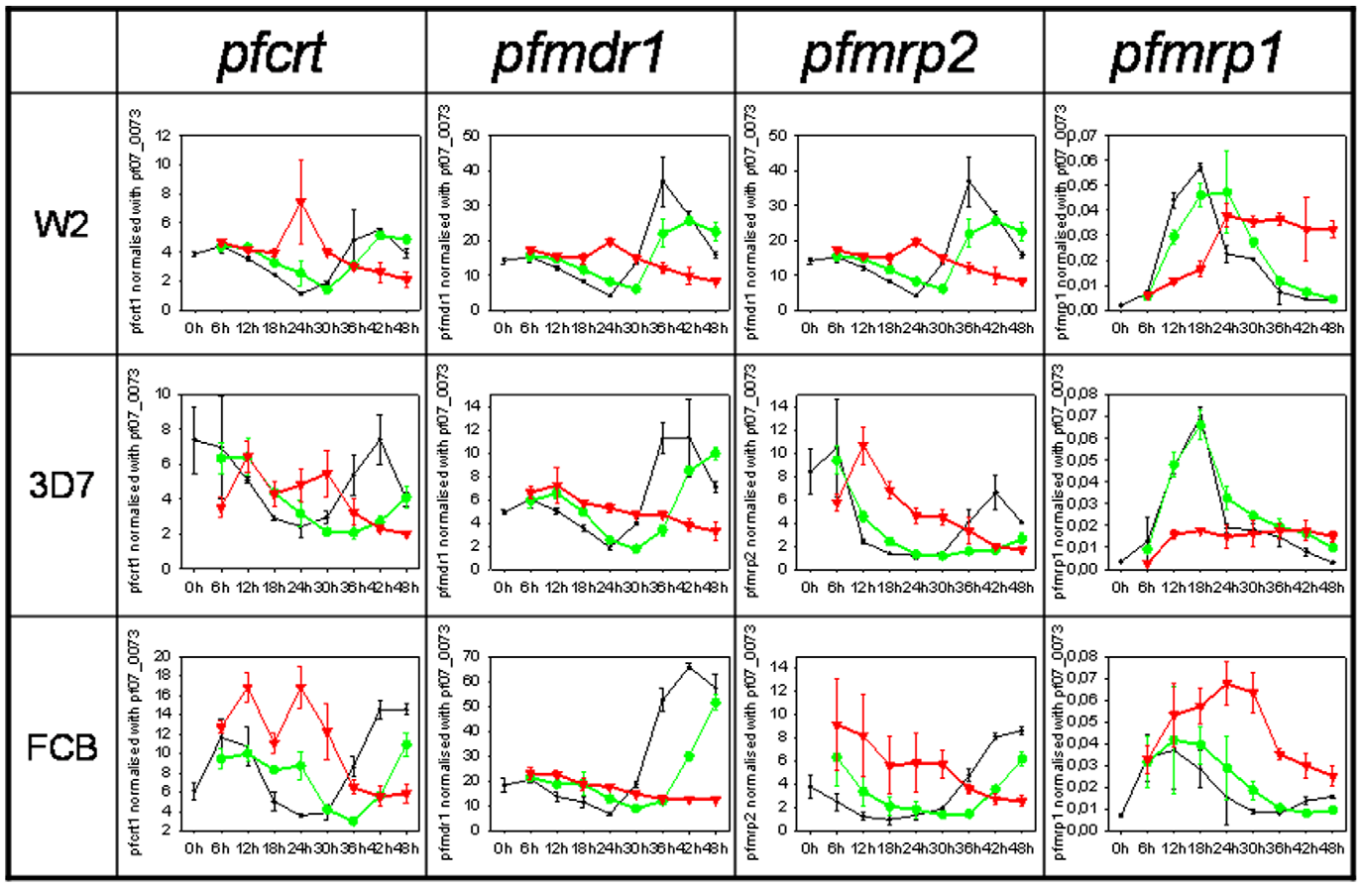
Drug transporter gene expression throughout time upon MQ exposure. Parasites cell cycle time course of drug transporters gene expression at basal level (control, black line) and after exposure to MQ IC_50_ (green lines) and MQ IC_99_ (red lines). X axis represent time-points in hours and Y axis relative gene expression quantification using pf07_0073 as endogenous control gene. Error bars represent standard deviation from 3 *in vitro* replicates.

**Figure 3:**
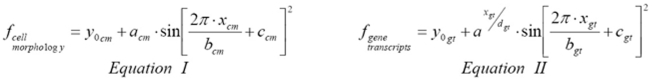
Non linear regression equation I (for cell morphology) and II (for gene transcripts). y_0_ = offset, corresponding to the initial (experimental t_o_) status of the parameter under analysis (e.g. number of parasites at ring stage); a = wave amplitude; b = wave period (the reciprocal wave frequency); b^−1^ = wave frequency; c = wave phase; x = time; d = damping constant factor (to account to dampening variations in amplitude of gene expression). _cm_: cell morphology; _gt_: gene transcripts.

**Figure 4:**
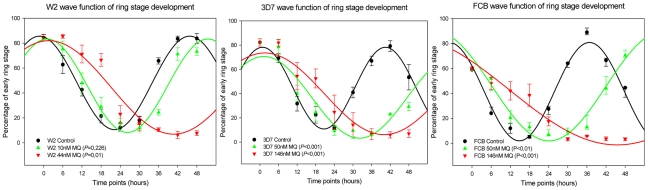
Effect of MQ in *P.falciparum* stage development. Wave form smoothing function best fitted over the early ring stage cell count (Figure 1, brown color). The wave frequency value (time needed to complete a cell cycle) for each fitted function (control, MQ IC_50_ and IC_99_) was used for the ratio calculation delay between treated and non treated parasite cultures (Table 1). Statistical comparisons (t-tests) of treatment effects (control *vs.* MQ IC_50_ and IC_99_) were performed with the associated error of wave frequency value of different experiments per clone compared with control.

Each gene has its own stage morphology expression profile, which once induced by drugs, it was previously shown that the expression is strictly correlated with the cell morphology [26], [27]. The MQ induced cell morphology delay was also detectable through the analysis of the transcript accumulation patterns (Figure 2, green and red curves). Applying the described non-linear regression model to the cycle (figure 3), significant differences in the degree of cell morphology progression were seen with MQ IC_99_ exposure in the three clones (p<0.05; figure 4: red lines). The calculated rate at IC_50_, compared to control was clone dependent. The effect was less pronounced in W2 parasites with approximately 15% delay (p = 0.23) *vs.* ∼40% for the MQ tolerant clones FCB and 3D7 (p<0.01) (Figure 4 and Table 1). Similar cell cycle morphology delay events were also observed upon quinine exposure (IC_50_ and IC_90_) in exploratory 12 hour follow up assay (Figure S2).

**Table 1:** Cell cycle development and gene expression of parasites challenged with MQ IC_50_.

Clones	Cell cycle progression%* (SE)	*Pfcrt*%† (SE)	*pfmdr1*%† (SE)	*pfmrp2*%† (SE)	*pfmrp1*%† (SE)
W2	85.3 (12.9)	91.5 (5.6)	107.9 (9.5)	81.2 (9.8)	104.9 (11.8)
3D7	64.3 (8.1)	56.6 (16.6)	98.8 (6.4)	96.4 (14.9)	107.3 (22.9)
FCB	61.2 (10.4)	86.1 (6.3)	109.8 (11.2)	107.5 (26.8)	77.5 (24.5)

* - Cell cycle development percentage was calculated as the ratio of the time needed to complete a cycle obtained from the fitted wave function (Figure 3, equation I) of control and MQ IC_50_ experiments (Figure 4).

† - Gene expression percentage was calculated as the ratio of the time needed to complete a cycle obtained from the fitted damped wave function (Figure 3, equation II) of control and MQ IC_50_ experiments (data not shown).

### Basal gene expression of transporter genes in W2, 3D7 and FCB

The *pfmdr1*, *pfcrt*, *pfmrp1* and *pfmrp2* cell cycle patterns of relative transcript abundance from control (i.e. non drug exposed) experiments for the reference 3D7 clone were similar to the available data at PlasmoDB [28] (Figure 2, black curves), using as endogenous control *seryl*-tRNA *synthetase* gene. In brief, *pfmdr1*, *pfcrt* and *pfmrp2* displayed the lowest levels of expression at approximately 24 hours (middle/late trophozoite stage) and highest amplitudes around 42–48 hours post invasion. *pfmrp1* revealed the previously described clearly different expression profile [29], [30], with a peak of expression during the development of trophozoites (12–24 hours post-invasion).

Comparing relative transcript abundance (R±SD) between the three clones at onset (Figure 2, time-point 0h), *pfmdr1* mRNAs showed higher levels in the FCB clone (18.15±2.84), as compared to W2 (14.18±0.99) and 3D7 (4.90±0.21) with a fold difference of 1.3 and 3.7 respectively. *Pfcrt* showed to be less expressed in the W2 (3.86±0.15) compared with 3D7 (7.40±1.90) and FCB (6.10±0.88) with a fold difference of 0.5 and 0.63 respectively. As for the *mrp* genes, *pfmrp2* gene showed higher expression for 3D7 (8.40±1.95) compared with W2 (5.06±0.13) and FCB (3.81±1.03) with a fold difference of 1.7 and 2.2 respectively while *pfmrp1* at time-point 0 the results was 3.5 fold difference between FCB (0.007±9^E-4^) and W2 (0.002±1^E-4^) and 1.75 fold comparing with 3D7 (0.004±6^E-4^).

### Specific drug transporter genes induction by mefloquine

**Figure 5:**
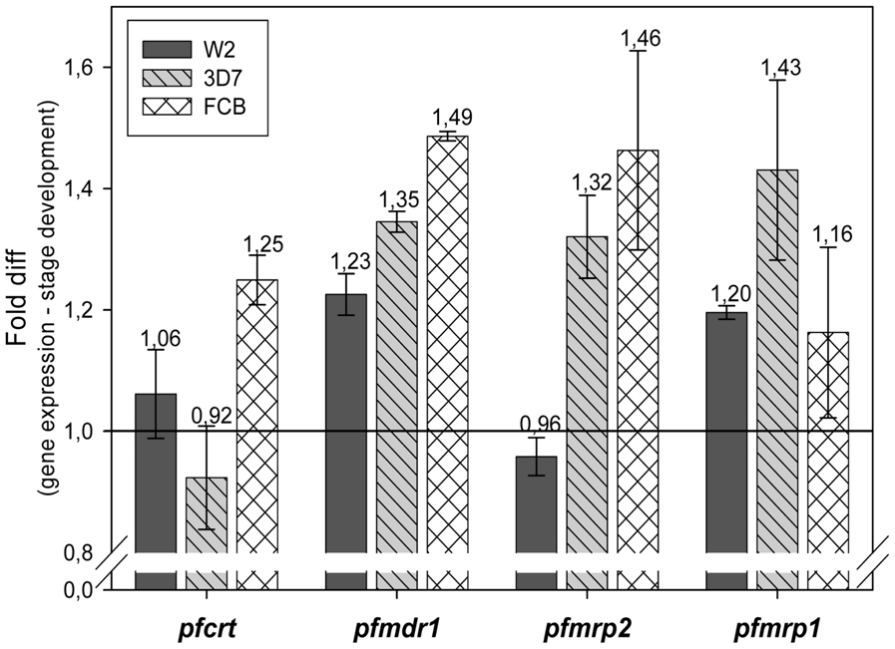
Drug transporter genes induction by MQ IC_50_. Results appear as fold difference in gene expression normalized with cell cycle stage development achieved after smoothing the data with wave functions. Error bars represents SE of the difference.

The fact that the drug exposure led to a proliferative delay in the parasites stage created a new challenge on how to differentiate gene transcription levels affected by cell morphology from the direct action of the drug on the transcript accumulation of the studied genes. We solved this challenge through two approaches: **(1)** From the few possible comparative time-points (control *vs.* MQ exposure time-points that had statistically non distinguishable parasite morphology proportion differences (p>0.05)), transcript abundance values were compared. The levels of significant gene expression modulation, within each clone upon MQ exposure, ranged from 0.6 to 5.8 fold: *pfmdr1* (0.6–1.5 fold), *pfcrt* (0.8–1.3 fold), *pfmrp2* (0.8–2.7 fold) and *pfmrp1* with the highest MQ driven induction (0.7–5.8) (Table 2). **(2)** To complement this limited comparative point by point verification of drug direct action, which gave rise to few analyzable points, another analytical approach based on a non-linear regression analysis of gene expression over time was conducted for the MQ IC_50_ experiments. The calculated fold difference confirmed that the drug exposure was generally associated with mild changes in the expression of the genes (figure 5). Considering each gene at IC_50_ exposure: (a) The *pfmdr1* gene, was significantly more induced in the less MQ sensitivity clones 3D7 (1.35 fold, p = 0.029) and FCB (1.49 fold, p<0.001), than in the more sensitive W2 (1.23 fold). Comparisons between the two less sensitive parasites showed that FCB can induce this gene significantly more than 3D7 (p = 0.003). (b) As for *pfmrp1*, our data show no statistical difference between the clones. (c) Concerning *pfcrt* gene, the W2 and 3D7 parasites appear to lack the capacity to significantly induce *pfcrt* upon MQ exposure (1.06 fold and 0.92 fold) compared to FCB (1.25 fold). (d) *Pfmrp2*, appears to significantly induce the less MQ sensitivity clones 3D7 (1.32 fold, p = 0.003) and FCB (1.46 fold, p = 0.023) compared with W2 (0.96 fold).

**Table 2:** Gene transcripts time points comparison in MQ exposed parasites with identical stage proportions.

Clones	Comparable time points†		Comparable time points Chi-square	*pfcrt*					*pfmdr1*					*pfmrp2*					*pfmrp1*				
**W2**	Control	IC_50_	X^2^	**Fold diff** *	diff mean	SD	t-test	P-value	**Fold diff**	diff mean	SD	t-test	P-value	**Fold diff**	diff mean	SD	t-test	P-value	**Fold diff**	diff mean	SD	t-test	P-value
	12	12	0.06	**1.2**	−0.8	0.2	−6.1	0.00*	**1.2**	−2.5	0.6	−5.2	0.01*	**1.5**	−1.1	0.1	−17.2	0.00*	**0.7**	0	0	6	0.00*
	24	30	0.08	**1.3**	−0.3	0.1	−4.7	0.01*	**1.5**	−2	0.3	−8.1	0.00*	1.3	−0.2	0.1	−2.4	0.08	1.2	0	0	−2.1	0.1
	36	42	0.11	1.1	−0.3	1.5	−0.3	0.81	**0.7**	11.4	5.1	2.8	0.05*	1.1	−0.4	0.4	−1.5	0.22	1	0	0	0	1
	36	48	0.09	1	−0.1	1.5	0	0.97	**0.6**	14.4	5.4	3.3	0.03*	1.2	−1	0.5	−2.4	0.07	0.6	0	0	1	0.38
	Control	IC_99_																					
	0	6	0.95	**1.2**	−0.8	0.2	−5.4	0.01*	**1.2**	−3	0.9	−4.1	0.02*	**0.9**	0.7	0.2	5	0.01*	**3.2**	0	0	−17	0.00*
	6	18	0.5	0.9	0.5	0.4	1.5	0.2	1	0.4	1.6	0.3	0.79	1.2	−0.8	0.5	−1.9	0.13	**2.4**	0	0	−4.9	0.01*
	42	6	0.16	**0.8**	0.9	0.2	6.4	0.00*	**0.6**	9.8	1.1	11.4	0.00*	**0.8**	0.8	0.2	5.9	0.00*	**1.3**	0	0	−5.4	0.01*
	48	6	0.58	**1.2**	−0.8	0.3	−3.8	0.02*	1.1	−1.4	0.8	−2.1	0.1	1	0.1	0.2	0.7	0.52	**1.6**	0	0	−8.5	0.00*
**3D7**	Control	IC_99_																					
	42	6	0.43	0.5	0.7	2.1	0.4	0.72	0.6	−0.2	0.6	−0.4	0.71	0.9	3.2	3.1	1.2	0.28	0.4	0	0	−1.9	0.13
	48	12	0.64	1.6	0.2	0.7	0.3	0.79	1	−0.5	0.6	−1	0.37	**2.7**	−1.2	0.4	−3.8	0.02*	5.2	0	0	−1.8	0.15
	48	18	0.65	**1.1**	3.2	1.1	3.7	0.02*	0.8	4	2.4	2	0.11	1.7	0.3	1.1	0.3	0.76	**5.8**	0	0	4.4	0.01*
**FCB**	Control	IC_50_																					
	6	12	0.09	0.9	1.7	1.5	1.4	0.24	0.9	2.1	1.4	1.9	0.14	1.3	−0.9	1	−1	0.37	1.2	0	0	−0.5	0.63

*: Fold difference is represented in bold for the significant (t-test, p<0.05) gene expression MQ induction.

† : For each clone, the four morphological stages count in all time points from control experiments and MQ exposed were compared (Chi-square). Only the time points with same proportion of stages (no statistical difference, p>0.05) are included in the table for MQ gene expression induction analysis with different ICs.

## Discussion

Upon continuous MQ exposure we have documented a dose dependent delay of the parasite cell cycle, particularly among the two MQ less sensitive *P. falciparum* clones (3D7 and FCB). This observation prompts the discussion of its biological importance in terms of drug resistance response.

Mechanistically, the phenomenon of drug resistance is essentially associated with a decrease in the number of effective opportunities for the therapeutic drug to effectively engage to its target(s). Below a certain threshold of interaction rate, the pharmacodynamic effect of the drug will be reduced to a degree that makes it clinically ineffective (hence associated to a treatment failure). This can be achieved in the cell by decreasing drug concentration in the target cellular compartment through the action of transmembrane transporters. To this a reduced availability of the target can be added, particularly through a decrease in metabolic activity, potentially related with a slow down of the cell cycle.

In several clinically relevant biological systems – notoriously in cancer cells - drug exposure leads to a noticeable slowing down in cell cycle progression, even to the point of stalling [31], [32]. In parallel, drug driven cell cycle arrest in *P. falciparum* has been consistently described by us and others for several antimalarial drugs [6], [7] including MQ [8] and very recently chloroquine [33]. *P. falciparum* dormant states are essentially refractory to antimalarial action [6] presumably due to its low metabolic activity status. Upon these observations, we speculate that the observed cell cycle delay behavior might functionally constitute a first stage towards this dormant state. The observed capacity of the different parasites clones to full recover growth, after 48 hours of intense MQ IC_99_ exposure, in different recrudescence time indirectly supports this suggestion.

The capacity to delay the intra-erythrocytic developmental cycle could therefore be an operational contributor for drug resistance, particularly when considering drugs with short half-lives, like artemisinins and quinine. Such a response could help the parasite to withstand the effects of the drug at its peak serum concentration, increasing the chances of survival as the drug concentration rapidly decreases in the circulatory system, due to elimination. The delay in the cycle might also be of importance for allowing time for a parallel action: the specific induction of drug transporters able to reduce the intra-cellular (or intra-compartment) concentrations of the drug. This action can in turn gain time for the process of metabolic shut off leading to a drug action refractory state based on the scarcity of drug targets.

Our results show a gene expression induction of generally less than 2 fold after MQ challenge, suggesting that these genes are actually inducible by this drug, though to a limited extent. Similar induction levels have been observed for *pfmdr1* (1.7 fold, when normalized for *hsp86*) in a northern blot based approach, after a 6h MQ treatment [34]. This data is also coherent with a trend of low *pfdhfr* gene transcriptional response previously reported upon exposure to the experimental antifolate WR99210 [26], as well as to chloroquine [27]. It is to note that the observed relatively low induction of transcription seen in response to MQ does not exclude more significant responses upon exposure to other xenobiotics. Accordingly, treatment of the *P. falciparum* clone 3D7 with phenobarbital (a classical drug elimination inducer) for 48h was associated to an extensive increase in *pfmdr1* transcripts, and to a concordant reduction in drug susceptibility [35].

These observations raise the question on how important mild changes in gene expression can be in terms of the parasite response to the drug. When drug resistance is mainly based on direct modifications of the target, as in the case with antifolates, an increase in drug quantity of the latter will probably be irrelevant, since it is unlikely that the number of drug molecules will overcome the number of target proteins (considering a regular stoichiometric relationship of action). In the case of transporter proteins - assuming that they are not themselves targets for the drug - the situation is markedly different. One protein can transport a large number of molecules per time unit, and a simple 2 fold increase in this capacity may well have an impact on the effect of the drug. This is supported by *in vivo* and *in vitro* studies showing that a two fold gene copy number amplification of *pfmdr1* significantly affects the parasite response to MQ and other drugs [22], [23], [36], [37]. Interestingly, the clones herein observed with the largest induction of transporter genes transcripts in response to MQ were also the less sensitive to the drug (3D7, FCB), suggesting a role of increased expression in the overall parasite drug response.

Our observations of significant parasite cycle delay upon drug exposure points this as a significant confounding factor in the analysis of parasite drug driven gene expression. Hence, we propose a simple non-linear regression model as a novel analytical tool to distinguish drug specific gene induction from the effect of the time changed gene expression variation associated to the cell cycle. This approach could be applied in future studies, as well as revisiting previous data that did not consider the phenomenon of drug driven cell cycle delay in *P. falciparum*.

In conclusion, MQ is able to induce expression of *pfmdr1*, *pfcrt*, *pfmrp1* and *pfmrp2* which, although mild, are potentially significant in aiding the parasite to evade antimalarial drug action. This occurs in the context of a prompt MQ driven cell cycle delay that potentially constitutes a further parallel mechanism for the parasite populations to avoid, or at least delay, antimalarial action.

## Materials and Methods

### *P. falciparum* parasite clones

*P. falciparum* 3D7 clone was obtained from Prof. D. Walliker (Department of Animal and population genetics, University of Edinburgh, UK), while parasite clones W2 (MRA-157) and FCB (MRA-309) were obtained from Malaria Research and Reference Reagent Resource Center (MR4, ATCC Massanas Virginia). The parasite lines were selected on the basis of their sensitivity to mefloquine hydrochloride (Sigma-Aldrich®, St.Louis, MO, USA) and their genetic background (sequences submitted to GenBank accession numbers: GU797309, GU797310, GU797311, GU797312, GU797313, GU797314, GU797315, GU797316).

### Drug susceptibility determinations

The inhibitory concentration of 50 and 99% for the three clones was assessed using an Histidine-Rich Protein 2 based Double-Site Sandwich Enzyme-Linked Immunosorbent Assay [38] followed by nonlinear regression analysis (http://malaria.farch.net).

### *P. falciparum* in vitro culture

The parasites were kept in culture using conventional methods [39] in human O^+^ RBCs and Malaria Culture Medium, containing RPMI 1640 culture medium supplemented with 10% L-glutamine, 0.05% gentamicine (Gibco®/Invitrogen™, Carlsbad, CA, USA) and 10% human AB^+^ serum. Large scale culture of each clone was produced with 3% parasitaemia in 2.5% hematocrite and synchronized in early ring stage at least twice by applying the MACS® system (Miltenyi Biotec, Bergisch Gladbach, Germany). The batch was then divided for three experiments: one control culture (no drug added) and two cultures with MQ IC_50_ and IC_99_.concentration followed by spreading it in culture plates (2mL/well). At the time of reinvasion, early rings (time-point 0), two Giemsa-stained smears were made for examination of parasites stages, while three replicate wells of parasite culture were harvested for RNA extraction. This procedure was followed for the three experiments, in six hour time-point intervals up to 48 hours (total of 9 time-points analyses including time 0). The smears were fixed with methanol and stained with 5% Giemsa and visualization under light microscopy (1000× magnification). Their examination included counting 100 parasites, twice per slide (2 slides per time point), distinguishing four main morphological stages: early rings, late rings/early trophozoites, trophozoites and schizonts. A total count of 400 parasites per time-point, per experiment was performed. The experimental design was equally applied for the three clones analyzed (W2, 3D7 and FCB).

### Molecular analysis

Parasite cultures collected for RNA extraction were centrifuged at 0.8 g-force for 2 min. 1.85 mL of supernatant were removed and 150 µL of PBS plus 300 µL of Lysis buffer (Applied Biosystems™, Fresno, CA, USA) were added. The mix was kept at −20°C until RNA extraction. RNA extraction was carried out using an ABIPRISM®6100 Nucleic Acid PrepStation® (Applied Biosystems™, Fresno, CA,USA) according to the recommendations of the manufacturer. Total RNA quality and quantity was measured using the AgilentRNA 6000 Pico total RNA assay in an Agilent2100 Bioanalyser™ (Santa Clara, CA, USA). Sample concentrations with RNA Integrity Number >5 were normalized for each clone before cDNA synthesis (High-Capacity cDNA Reverse Transcription Kit (Applied Biosystems, Fresno, CA, USA)). Real-time analyses were performed in an ABIPRISM® 7900HT Sequence Detection System (Applied Biosystems™, Fresno, CA, USA) with gene specific MGB TaqMan® probes and primers. TaqMan® probes and primers for target gene *pfmdr1* were as previously published [22]. New designs were developed for *pfcrt*, *pfmrp1*, *pfmrp2* and for endogenous control gene [30], [40] *seryl*-tRNA *synthetase* (PF07_0073) (Table S1). Amplification reactions were done in quadruplicate in 384 well plates with 10µl containing TaqMan® Gene Expression Mastermix (Applied Biosystems™, Fresno, CA, USA), 300nM of each forward and reverse primer, 100nM of TaqMan®probe and 2 µL of amount-normalized cDNA. The thermal cycle profile was 50°C for 2 min, 95°C for 10 min and forty cycles of 95°C for 15 s and 60°C for 1 min. The amplification efficiency (E) was estimated for each gene and used as a correction factor for relative gene expression quantification. All experimental threshold cycle values (Ct) were first transformed to adjust the RNA concentration adding to the Ct value the log_2_ RNA concentration of each clone. Relative gene expression was calculated as the ratio (R) between the transformed Ct values of target gene and internal control reference gene (PF07_0073), taking in account the amplification efficiency for each gene [41]. Calculations were conducted using the SAS 9.1 system for Windows™.

### Determination of the drug exposure specific induction effect: time-point gene expression fold differences and the non-linear regression model approach

In order to distinguish the changes in cDNA abundance associated with the parasite cell cycle progression from the ones associated with drug exposure, two different approaches were performed:

#### I. Direct comparisons

Fold difference in transcript abundance was calculated in control *vs.* MQ exposed cultures only in time-points showing a statistically equal fractional composition of the four microscopically determined stages (Fisher test, df = 3, p>0.05).

#### II. Non linear regression analysis

A wave function applied to the number of total parasites ring stage count throughout all time-points for each clone (**equation I** – figure 3) was achieved (goodness of fit was R^2^>0.9) (SigmaPlot™ 9.0). Specific parameters of this function were used to quantify the parasite delays in cell morphology (_cm_) imposed by MQ exposure. The wave frequency (b_cm_^−1^) from each fitted function (control, MQ IC_50_ and MQ IC_99_ exposed), corresponds to the time needed to complete a cycle and it was used to calculate the cell cycle delay based in the observation of the morphology development of the parasite between MQ exposed (IC_n_) and control (R_morphology_ = b_cm_^−1^_ICn_/b_cm_^−1^_control_). Statistical comparisons (t-tests) were performed with the associated error of the wave frequency parameter “b_cm_^−1^”.

A similar approach, of applying a wave function, was performed in the gene transcripts data (_gt_). During the 48 hours exposure to MQ IC_50_, the associated delay in the cycle gene expression was calculated by comparing the wave frequency parameter “b_gt_^−1^” (R_gene transcripts_ = b_gt_^−1^_IC_/b_gt_^−1^_control_) obtained through a best fit of the gene expression data, this time in a damped wave function (**equation II** – figure 3). Statistical comparisons (t-tests) were performed with the associated error of the wave frequency parameter “b_gt_^−1^”.

The use of these equations allowed us to determine whether the expression profiles were solely stage-driven, or included a significant fraction associated with drug exposure:
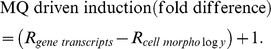
By its nature, this approach was only possible to apply to experimental outputs where sufficient data points were available for enabling fitting a sine function, being hence only applicable to gene transcripts data concerning IC_50_ and not to the IC_99_ drug level exposure.

## Supporting Information

Figure S1 Cell viability after MQ IC_99_ exposure. Parasite strains viability after challenged with continuous MQ IC_99_ (W2, 44nM; 3D7 and FCB, 146nM) for 48 hours analyzed by Histidine-Rich Protein 2 Double-Site Sandwich Enzyme-Linked Immunosorbent. All strains had a growth recovery of 7–10 days after drug withdrawal. Error bars are SE of rate between day0 HRP2 and collected day.
(0.06 MB PDF)

Figure S2 Cell cycle morphology progressions upon quinine exposure (IC_50_ and IC_90_) after 12 hour follow up. Parasite stage percentage (Y axis) of W2, FCB and 3D7 strains after 12 hours (X axis) with continuous exposure of quinine IC_50_ and IC_90_. The counting of the parasites included 100 parasites, twice per slide (2 slides per different IC_s_ exposure), distinguishing four main morphological stages using light microscope: early rings, late rings/early trophozoites, trophozoites and schizonts.
(0.01 MB PDF)

Table S1 TaqMan® probes and primers sequence.
(0.03 MB DOC)
