# Supplementary material for: Antimalarial Exposure Delays Plasmodium falciparum Intra-Erythrocytic Cycle and Drives Drug Transporter Genes Expression
Source: PLoS One. 2010 Aug 25;5(8):e12408. doi: 10.1371/journal.pone.0012408 (PMC2928296; doi:10.1371/journal.pone.0012408)
Supplement: Figure S1 — Cell viability after MQ IC99 exposure. Parasite strains viability after challenged with continuous MQ IC99 (W2, 44nM; 3D7 and FCB, 146nM) for 48 hours analyzed by Histidine-Rich Protein 2 Double-Site Sandwich Enzyme-Linked Immunosorbent. All strains had a growth recovery of 7–10 days after drug withdrawal. Error bars are SE of rate between day0 HRP2 and collected day. (0.06 MB PDF) [file pone.0012408.s001.pdf]

Supplementary figure 1: Cell viability after MQ IC_{99} exposure

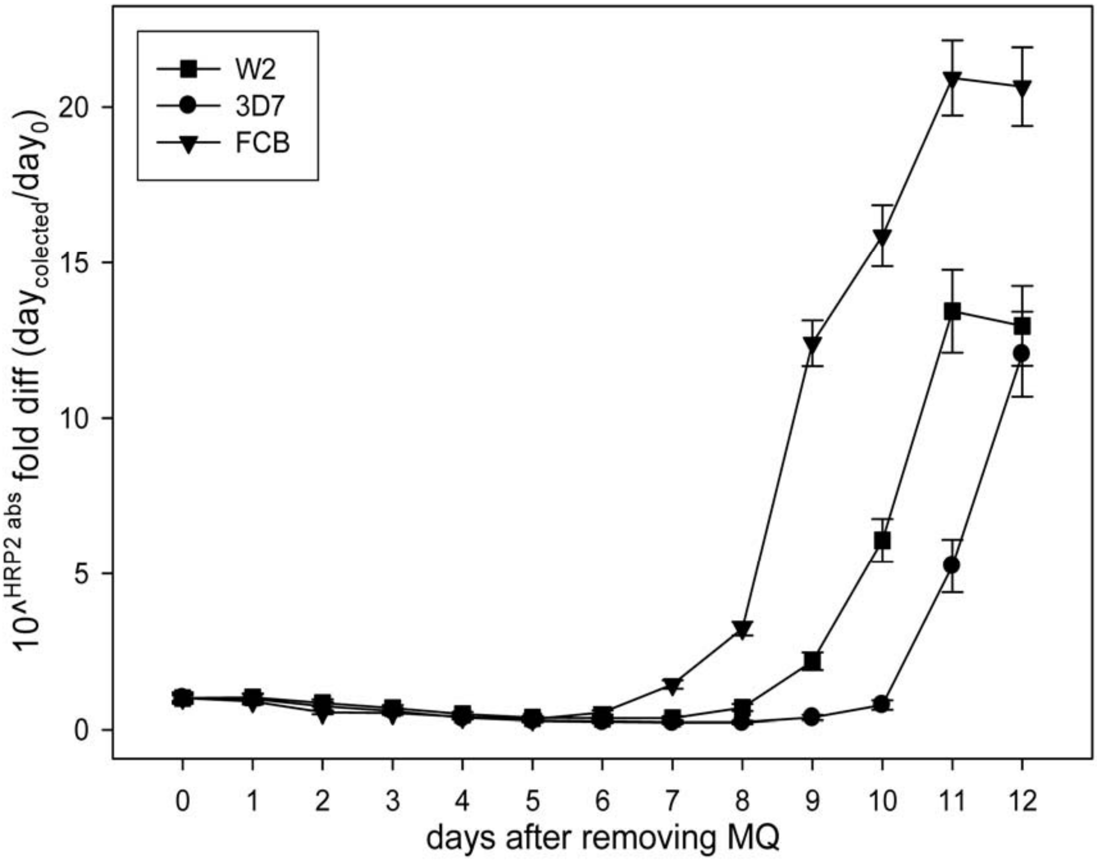

Parasite strains viability after challenged with continuous MQ IC_{99} (W2, 44nM; 3D7 and FCB, 146nM) for 48hours analyzed by Histidine-Rich Protein 2 Double-Site Sandwich Enzyme-Linked Immunosorbent. All strains had a growth recovery of 7-10 days after drug withdrawal. Error bars are SE of rate between day0 HRP2 and collected day.
