# Supplementary material for: Antimalarial Exposure Delays Plasmodium falciparum Intra-Erythrocytic Cycle and Drives Drug Transporter Genes Expression
Source: PLoS One. 2010 Aug 25;5(8):e12408. doi: 10.1371/journal.pone.0012408 (PMC2928296; doi:10.1371/journal.pone.0012408)
Supplement: Figure S2 — Cell cycle morphology progressions upon quinine exposure (IC50 and IC90) after 12 hour follow up. Parasite stage percentage (Y axis) of W2, FCB and 3D7 strains after 12 hours (X axis) with continuous exposure of quinine IC50 and IC90. The counting of the parasites included 100 parasites, twice per slide (2 slides per different ICs exposure), distinguishing four main morphological stages using light microscope: early rings, late rings/early trophozoites, trophozoites and schizonts. (0.01 MB PDF) [file pone.0012408.s002.pdf]

Supplementary figure 2: Cell cycle morphology progressions upon quinine exposure (IC50 and IC90) after 12 hour follow up.

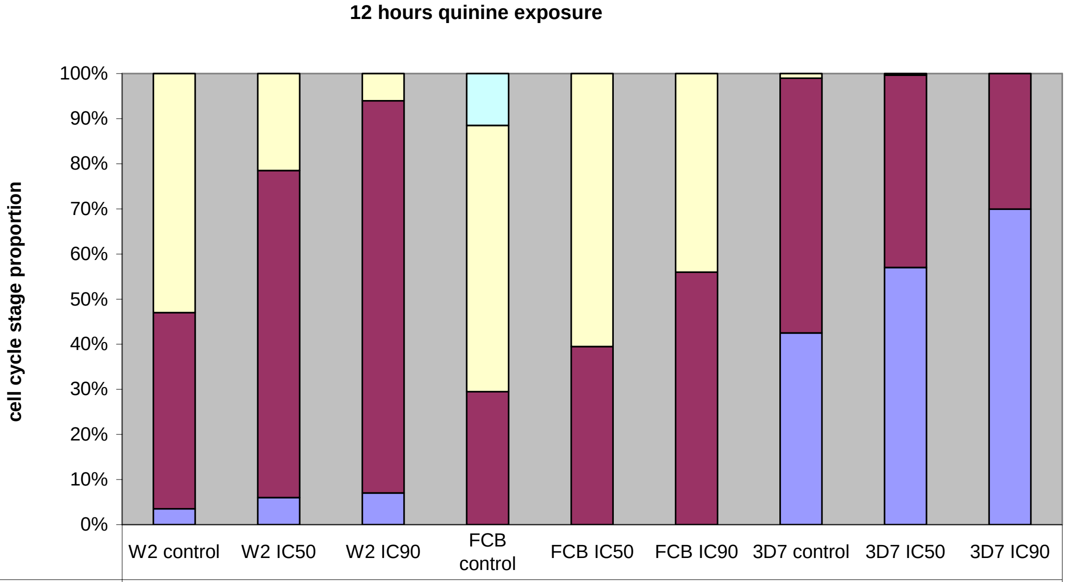

	W2 control	W2 IC50	W2 IC90	FCB control	FCB IC50	FCB IC90	3D7 control	3D7 IC50	3D7 IC90
schizont	0,00	0,00	0,00	0,12	0,00	0,00	0,00	0,00	0,00
troph	0,53	0,22	0,06	0,59	0,61	0,44	0,01	0,00	0,00
Late ring/early troph	0,44	0,73	0,87	0,30	0,40	0,56	0,57	0,43	0,30
Ring	0,04	0,06	0,07	0,00	0,00	0,00	0,43	0,57	0,70

Parasite stage percentage (Y axis) of W2, FCB and 3D7 strains after 12 hours (X axis) with continuous exposure of quinine IC_{50} and IC_{90}. The counting of the parasites included 100 parasites, twice per slide (2 slides per different IC_s exposure), distinguishing four main morphological stages using light microscope: early rings, late rings/early trophozoites, trophozoites and schizonts.
