# Supplementary material for: Antimalarial Exposure Delays Plasmodium falciparum Intra-Erythrocytic Cycle and Drives Drug Transporter Genes Expression
Source: PLoS One. 2010 Aug 25;5(8):e12408. doi: 10.1371/journal.pone.0012408 (PMC2928296; doi:10.1371/journal.pone.0012408)
Supplement: Table S1 — TaqMan® probes and primers sequence. (0.03 MB DOC) [file pone.0012408.s003.doc]

**Supplementary Table 1: TaqMan®** probes and primers sequence

| **Gene name** | **Gene ID** | **Probe** | **Primers** |
| --- | --- | --- | --- |
| *pfmdr1* | PFE1150w | **6-FAM, TAMRA probe** | Fw TGCATCTATAAAACGATCAGACAAA  Rev TCGTGTGTTCCATGTGACTGT |
|  |  | GTATTTAATAACCCTGATCGAAATG  GAACCTTTG |  |
| *pfcrt* | MAL7P1.27 | **6-FAM, MGB probe** | Fw CGACACCGAAGCTTTAATTTACAAT  Rev AAGACCTATGAAGGCCAAAATGAC |
|  |  | CTATATCCATGTTAGATGCC |  |
| *pfmrp1* | PFA0590w | **6-FAM, ABI custom gene expression assay** | FwTCAGGATAAAACTTTAAAATATAGAGGAAATATATCAGAATATATGGA  Rev GTTCCACTTCATCAAACAATTTTATTCTGGTA |
|  |  | ACCCCAGTGACTTTCT |  |
| *pfmrp2* | PFL1410c | **6-FAM, MGB probe** | Fw AATAATGACCCTACCCACGAAGA  Rev CATACGAACCAGGAACAGATTTAAA |
|  |  | AGCAGGATGCAAATT |  |
| *pfseryl-tRNA synthetase* | PF07_0073 | **VIC, TAMRA Probe** | Fw CCTCAGAACAACCATTATGTGCTT  Rev TGTGCCCCTGCTTCTTTTCTA |
|  |  | TGAAACTATAGAATCAAAAAGGTT  ACCACTCAAATACGCT |  |
